# The expression of vulnerability through infant mortality in the municipality of Embu

**DOI:** 10.1590/S1516-31802008000500004

**Published:** 2008-09-04

**Authors:** Renato Nabas Ventura, Rosana Fiorini Puccini, Nilza Nunes da Silva, Edina Mariko Koga da Silva, Eleonora Menicucci de Oliveira

**Keywords:** Infant mortality, Social class, Health priorities, Child, Health services accessibility, Mortalidade infantil, Classe social, Prioridades em saúde, Criança, Acesso aos serviços de saúde

## Abstract

**CONTEXT AND OBJECTIVE:**

Infant mortality expresses a set of living, working and healthcare access conditions and opens up possibilities for adopting interventions to expand equity in healthcare. This study aimed to investigate vulnerability and the consequent differences in access to health services and occurrences of deaths among infants under one year of age in the municipality of Embu.

**DESIGN AND SETTING:**

This was a descriptive study in the municipality of Embu.

**METHODS:**

Primary data were collected through interviews with the families of children living in the municipality of Embu who died in the years 1996 and 1997 before reaching one year of age. Secondary data were obtained from death certificates. The variables collected related to living conditions, income, occupation, prenatal care, delivery and the healthcare provided for children. These data were compared with the results obtained from a study carried out in 1996.

**RESULTS:**

Statistically significant differences were found with regard to income, working without a formal employment contract and access to private health plans among the families of the children who died. There were also differences in access to and quality of prenatal care, frequency of low birth weight and neonatal intercurrences.

**CONCLUSIONS:**

The employment/unemployment situation was decisive in determining the degree of family stability and vulnerability to the occurrence of infant deaths, in addition to the conditions of access to and quality of healthcare services.

## RESUMO

**CONTEXTO E OBJETIVO:**

A mortalidade infantil expressa uma conjunção de fatores relacionados às condições de vida, trabalho e acesso aos serviços de saúde, e a identificação desses fatores pode contribuir para definição de intervenções em saúde. O objetivo deste trabalho foi analisar a expressão da vulnerabilidade e conseqüentes diferenças de acesso aos serviços de saúde e na ocorrência de óbitos em menores de um ano no município do Embu.

**TIPO DE ESTUDO E LOCAL:**

Estudo descritivo, no município de Embu.

**MÉTODOS:**

Foram coletados dados secundários (declarações de óbitos) e primários (entrevistas a famílias de crianças residentes do município do Embu, falecidas nos anos de 1996 e 1997, antes de completarem um ano). Variáveis estudadas foram relacionadas às condições de vida, renda e trabalho, à assistência pré-natal, ao parto e à atenção à saúde da criança, as quais foram comparadas com resultados obtidos em estudo realizado no ano de 1996.

**RESULTADOS:**

Verificaram-se diferenças estatisticamente significantes quanto a renda, trabalho sem carteira assinada e acesso a plano privado de saúde entre famílias de crianças que foram ao óbito. Verificaram-se, também, diferenças quanto ao acesso e à qualidade da assistência pré-natal, à freqüência de baixo peso ao nascer e a intercorrências neonatais.

**CONCLUSÕES:**

A situação de emprego/desemprego foi decisiva na determinação da estabilidade familiar, conferindo maior vulnerabilidade para ocorrência de óbitos infantis, somada às condições de acesso e à qualidade dos serviços de saúde.

## INTRODUCTION

Because infant mortality expresses a set of living, working and healthcare access conditions, it is one of the indicators used in studies dealing with social inequalities and seeking to comprehend the complex determination of the health-illness process. At the same time, the results from studies on infant mortality open up possibilities for using the information to adopt interventions that aim to expand equity in healthcare.^1^

The historical variability in infant mortality levels that has occurred because of institutional and socioeconomic transformations has resulted in changes to the determinants, intensities and order of importance of infant mortality. This phenomenon can be seen from analyzing historical series of infant mortality in developed countries. In these, the marked decreases in infant mortality over the last hundred and fifty years are related to the application of new technologies in the biomedical field and to structural changes in society caused by the industrial revolution and changes in the role of children in the family and in society.^2^

Although the reduction in mortality rates among children under five years of age in Latin American countries, including in Brazil, is the result of improvements in their real health situation, this reduction does not reveal the profound and persistent social inequalities between countries, between regions in each country and between different segments of their populations. Social inequity and poor living conditions constitute the principal barriers to advances and improvements in the whole population's health situation.^3^

In Embu, a municipality in the Metropolitan Region of São Paulo, the infant mortality rate was one of the highest in the State of São Paulo until the middle of the 1990s. It presented significant falls from 1996 onwards (when it was 28.1 per thousand live births), to reach 11.2 in 2006.^4^ This decrease occurred in relation to both neonatal and post-neonatal infant mortality. Neonatal deaths were responsible for around 60% of all deaths among children under one year of age, and the early neonatal component was responsible for around 40%.^4^ The fertility rate decreased from 130 per 1000 women aged 15 to 49 years, in 1980, to 64 in 1997.^4^ This, together with the better access among women and members of their families to information, and the creation of a prenatal care structure for at-risk cases within the primary healthcare network of Embu, in 1996, partially explains the observed neonatal mortality trend. Other factors may also have contributed towards the reduction in the neonatal and post-neonatal components of infant mortality in this municipality: expansion of the primary healthcare services and the diagnostic and therapeutic support; development of a regular program of full healthcare for children (which among other actions provides disease prevention and timely care for the most prevalent illnesses, particularly respiratory infections); and in 1999, the creation of a general referral hospital for medium and high-risk pregnancies with a neonatal intensive care unit and a pediatric ward, in this region.

Even though this reduction in infant mortality seen in Embu over this period can be considered positive, it is of fundamental importance to gain more in-depth knowledge about these deaths in the light of economic crises, unemployment and the persistence of inadequate environmental and housing conditions among a large proportion of the population. It has to be taken into consideration that differences may exist within this population, and thus the segments in situations of greater vulnerability need to be identified.

## OBJECTIVE

The present study had the objective of analyzing the expression of this vulnerability and the consequent differences in access to health services and occurrences of deaths among infants under one year of age in the municipality of Embu.

## METHODS

### 1. Characterization of the municipality of Embu and its health services

The municipality of Embu is 25 km from the city of São Paulo (the state capital), in the southeastern subregion of the Metropolitan Region of São Paulo. It has low concentrations of industrial activity and low economic growth potential, and large areas of the municipality are in water catchment protection areas.^5^ The municipality is a designated tourist destination, 100% of its urban area has been developed and in 1996 its population was 194,879 inhabitants.^4^ In 1996/1997, the period covered by this study, the municipal healthcare network consisted of nine primary healthcare units and two emergency services. There were no hospital wards. The Teaching/Attendance Integration Program of the Universidade Federal de São Paulo (PIDA-Embu/Unifesp), which had been under development in the municipality since 1970, involved the departments of Pediatrics, Neurology, Psychiatry, Gynecology, Obstetrics, Speech Therapy and Ophthalmology at the time of this study. In addition to teaching, attendance and research activities, the University had had active participation in the Municipal Health Council since 1991.^5^

### 2. Population and type of study

This was a descriptive study. From secondary data (death certificates), all deaths that occurred in the years 1996 and 1997 among children under one year of age who were living in the municipality of Embu were examined. The variables considered in this study were based mainly on the set of variables used in a previous project, "Infant morbidity and health service utilization", which had been funded by the Research Support Foundation of the State of São Paulo (Fundação de Amparo à Pesquisa do Estado de São Paulo, Fapesp).^6^ This set of variables related to living conditions, income, occupation, prenatal care, delivery and the healthcare provided for children. For the present study, other variables that were considered to be more directly related to the outcome (death) were added to this list. The method (sampling and data collection) for the project "Infant morbidity and health service utilization" is detailed in the report on the original project^6^ and in a study by Frei^7^ and will be summarized in the following.

This was a household survey that studied the population of children under five years of age who were living in the municipality of Embu in 1996, and also their families. According to the demographic census of 1991, which was used for calculating the sample size, this segment of the population was composed of 18,602 children. The approach taken for this project was based on analyzing situations of collective risk and assessing the intra-urban differentials through constructing composite indicators for studying the living conditions in the different areas. With data files available from the demographic census of 1991, the population was stratified according to the families' living conditions and socioeconomic situations. The 135 census tracts of the municipality were gathered together by means of a grouping analysis technique called "average linkage".^7^ The type of housing, the number of people living in each home, the type of basic sanitation and the head of the household's income and schooling level were the variables taken into consideration for discriminating between the resulting four strata: stratum 1 (which was concentrated in the touristic area of the municipality and presented the best environmental conditions, income, housing and schooling), strata 2 and 3 (which reflected the intermediate environmental conditions that predominated in the municipality); and stratum 4 (which included all the homes that were classified as substandard agglomerates or shantytown-type, with the worst environmental conditions, income, housing and schooling).

Two independent populations were considered for observation and analysis: children who had completed up to eleven months of age and children from 12 to 59 months of age. The limit tolerated for errors in the sample results obtained for each group was set at 5%, while the acceptable error for the estimates calculated for each stratum was set at 10%. The draw was carried out as a two-stage cluster process. In the first stage, 10 census tracts were drawn for each stratum, under the criterion of allotment proportional to their sizes,^8^ considering the number of homes registered in the 1991 census. In the second stage, homes in each previously selected census tract were drawn, after updating the number of homes that existed. The final sample was formed by 483 children under one year of age and 616 children from one to four years old. There were 475 families corresponding to the children under one year of age because fourteen of the children were twins and one family had two children under one year of age who were not twins. For comparison with the present study on deaths, only the group of children under one year of age was taken into consideration. Variables with more than 20% loss of information were not analyzed.

The data collection in the homes was carried out between August and November 1996 (morbidity project) and between August and November 1998 (families of children under one year of age who had died). For both of these studies, the information was obtained from the children's mothers whenever possible. After three visits, if no one capable of providing this information was found, the questionnaire was discarded. With regard to the prenatal and delivery data, only interviews held with the mothers were taken into consideration. The interviews were conducted by medium or higher-level health professionals, following training. There were 217 deaths during this period and 189 interviews were held: 119 with members of the families of children who died during the neonatal period and 70 with members of the families of children who died during the post-neonatal period. The loss of 12.9% was fundamentally due to the urban characteristics of the municipality and the consequent difficulties in finding the addresses of the families of the children who had died.

All the families interviewed, in both studies, received explanations about the research objectives and signed a consent statement. The study was approved by the Research Ethics Committee of the Universidade Federal de São Paulo and by the Municipal Health Department of Embu.

### 3. Variables

For the present study on deaths, two categories were constructed from the position that the family occupied in the productive structure (work and income) and the social reproduction conditions (individual and collective consumption, and access to health services). Some variables that were added in this study were just to enable comparisons between the subgroups of neonatal and post-neonatal deaths and comparisons with the data in the literature.

#### 3.1. Work and income:

The variables used were the number of people living in the home who were working, employment situations of the mother and the head of the household at the time of the child's death (when the mother did not have a partner, she was considered to be the head of the family), presence of a partner, family income expressed as multiples of the minimum monthly salary and access to private health insurance. This last variable was included in this category because of its relationship with salary level and/or formal employment contracts (through arrangements made by companies).

#### 3.2. Access to health services:

The access to prenatal care was deemed adequate when six or more consultations were attended, starting in the first trimester of pregnancy. The quality of the prenatal care was deemed to be adequate when laboratory tests were performed, arterial pressure measurements were made (at one or more consultations), breast examinations were performed (at one or more consultations) and at least one ultrasound examination was carried out (this examination formed part of the list of procedures in the municipality's prenatal care program). The prenatal care was deemed inadequate if one or more of these items were absent.

The delivery care variables consisted of the type of delivery, birth weight and neonatal intercurrences. Neonatal intercurrences were deemed to be present when they were mentioned in the interview and/or the discharge from the nursery occurred when the infant was more than five days old.

The use of health services by the children (at primary healthcare units or other locations) was considered with regard to the purposes of enrollment, follow-up (childcare), vaccination and consultations without appointments (for unexpected events).

### 4. Statistical analysis

The present study took into consideration all of the deaths that occurred in 1996 and 1997, and for this reason only the percentages were calculated. In the morbidity project, the Epi-Info software (version 6.04) was used, including the CSAMPLE module^9,10^ which allowed the data to be weighted. Different sampling fractions in each stratum introduced differences in the probability (*f*) that any family or child would belong to the sample that was drawn. For each stratum, the weight *w* was calculated as the inverse of the probability *f*. In other words, *w* = 1/f. The mathematical expression adopted for calculating italic resulted from the sampling plan selected.^6^

The statistical significance was assessed by considering the estimates, standard errors and 95% confidence intervals. The confidence interval obtained in the morbidity study was taken into consideration for comparisons with the percentages obtained in the present study on deaths, i.e. when the confidence interval of the morbidity study did not contain the percentage of the corresponding variable obtained among the children who died, it was considered that there was a possible difference between them (indicated in boldface).

## RESULTS

Table 1 shows the data relating to the category of work and income. It was found in both studies that only 60% to 70% of the families had one or more of their members in employment. Thus, the other families would evidently have been experiencing difficulties in relation to income. It is notable that 19.7% of the mothers in the families of the children who died during the post-neonatal period were performing the role of head of the family. Also in the same Table 1, it can be seen that the unemployment rates among the mothers of the children who died before completing one year ranged from 24.3% to 37.0%. This situation, added to the presence of other unemployed members of the family, would be a further determining factor for infant mortality. It is important to note that approximately 50% of the heads of households were working without formal employment contracts (both among the families in the morbidity project and among the families of the children who died during the post-neonatal period). Statistically significant differences were observed with regard to the number of people in the home who were working, *per capita* income and access to private health plans. Furthermore, the low percentage (13.6%) with access to private health insurance was notable among the families whose children died during the post-neonatal period, which is a reflection of their employment and income situation.

**Table 1.** Work and income characteristics of the families in the population survey (1996) and the families of children who died within their first year of life (1996-7) — Embu (SP)

Variables	Population survey				Families of children who died during the neonatal period (n = 119)*			Families of children who died during the post-neonatal period (n = 70)*		
	n	%	SE	95% CI	n	%	Total	n	%	Total
Number of people who were working (≤ 1)	297	64.7	2.2	**60.3-69.1**	79	**69.9**	113	46	**69.7**	**66**
Mother living with partner for less than 5 years					45	50.0	90	14	31.2	**44**
Mother working outside of home at the time of child's death	NA	NA	NA	NA	25	21.0	119	16	22.9	**70**
Mother working at home at the time of child's death	NA	NA	NA	NA	4	3.4	119	3	4.3	**70**
Mother unemployed at the time of child's death	NA	NA	NA	NA	44	37.0	119	17	24.3	**70**
Head of household working outside of home at the time of child's death	NA	NA	NA	NA	90	84.1	107	51	85.0	**60**
Head of household working at home at the time of child's death	NA	NA	NA	NA	4	3.7	107	2	3.3	**60**
Head of household unemployed at the time of child's death	NA	NA	NA	NA	8	7.5	106	9	15.5	**58**
Income ≤ 1 MMS (*per capita*)	226	53.5	7.2	**39.5-67.5**	62	54.9	113	42	**70.0**	**60**
Mother acting as head of household					11	9.7	113	13	19.7	**66**
Unemployment among other members of family					5	4.4	113	6	9.1	**66**
Head of household working without formal employment contract	203	48.2	6.2	**36.0-60.4**	25	**24.8**	101	28	47.5	**59**
Other income sources for family	58	10.4	1.8	6.8-14.0	14	12.5	112	5	7.7	**65**
Access to private health insurance	134	29.1	3.6	**22.0-36.2**	28	24.8	113	9	**13.6**	**66**
Median of total family income in MMS					5.0			4.2		

SE = standard error; CI = confidence interval; NA = not applicable; MMS = minimum monthly salaries.

* Information relating to the variables was not always available; the percentages were calculated by taking n as the number for which information was present.

Table 2 presents the data relating to the category of access to and quality of prenatal care and utilization of health services for follow-ups for the children. There were statistically significant differences in relation to the variables of access to prenatal care (starting in the first trimester and number of consultations greater than or equal to six) and quality of prenatal care, and also in relation to the subsequent follow-up for the children within the health service. With regard specifically to prenatal care, it was found that a higher percentage of the mothers of the children who died had had their prenatal care within the municipal network of Embu, which may reflect their lower access to private plans, as already presented in Table 1. Also in Table 2, it can be seen that there were statistically significant differences such that the group in which the children died was disadvantaged with regard to the frequency of neonatal intercurrences, especially with regard to the children who died during this period (95.5%) and those who presented low birth weight (63.9% and 36.9% of those who died during the neonatal and post-neonatal periods, respectively). The follow-ups within the municipal health services and other services also occurred with lower frequency among the children who died, even when considering only those who died during the post-neonatal period. Also in Table 2, the low percentage of mention of the hospital where the children who died had been delivered and the shuffling around, i.e. attendance provided in more than one hospital service during labor, reveal one of the serious deficiencies of the healthcare system regarding prenatal and delivery care. Among the 53 children who died after the neonatal period, in hospitals or emergency services, 24 of their families had sought the municipal services before the death. Of these, 50% were transferred to a more complex level of care and, in the cases of the other 50%, the families spontaneously sought the hospital where the child would eventually die. And in the cases of 14 of the 17 children who died at home during the post-neonatal period, the families said that they had sought medical attendance before the death, among which seven had sought the services of the primary healthcare network of Embu.

**Table 2.** Access to health services: population survey (1996) and subgroups of children who died during the first year of life (1996-7) — Embu (SP)

Variables	Population survey				Children who died during neonatal period (n = 119)*			Children who died during post-neonatal period (n = 70)*		
	n	%	SE	95% CI	n	%	Total	n	%	Total
Mother had prenatal consultations	451	96.3	1.2	**93.9-98.7**	103	**91.2**	113	60	**90.9**	**66**
Mother had prenatal consultations in Embu	247	54.4	3.1	**48.2-60.5**	66	**64.1**	103	43	**71.7**	**60**
Prenatal consultations started in first trimester	295	65.0	4.6.	**55.9-74.0**	43	**41.7**	103	24	**40.0**	**60**
Number of prenatal consultations ≥ 6	291	66.7	5.5	**55.8-77.5**	46	**46.5**	99	29	**51.8**	**56**
Adequate prenatal care (quality + access)	160	35.5	3.4	**28.8-42.1**	38	38.0	100	26	**44.8**	**58**
Delivery in hospital	463	97.7	1.1	**95.5-99.8**	121	**100.0**	121	66	98.5	**67**
Cesarean delivery	172	32.5	4.1	24.5-40.5	48	38.1	126	19	26.4	**72**
Neonatal intercurrences (discharge after fifth day)	44	7.5	2.1	**3.4-11.6**	106	**95.5**	111	11	**16.9**	**65**
Low birth weight (without twinning)	47	8.4	2.1	**4.3-12.5**	76	**63.9**	119	24	**36.9**	**65**
Enrollment in PHU	400	80.5	6.1	**68.6-92.3**	NA	NA	NA	38	**65.5**	**58**
PHU used for health follow-up	324	79.0	2.7	**73.8-84.2**	NA	NA	NA	31	**53.4**	**58**
PHU used for vaccination	409	97.8	1.1	**95.2-99.6**	NA	NA	NA	47	**81.0**	**58**
PHU used for possible consultations without appointment	230	91.1	2.8	**85.8-96.7**	NA	NA	NA	26	**44.8**	**58**
Health follow-up in other service	381	79.1	4.7	**69.9-88.3**	NA	NA	NA	30	**52.6**	**57**
Child's death occurred at home	NA	NA	NA	NA	2	1.7	121	17	25.8	**66**

SE = standard error; CI = confidence interval; NA = not applicable; PHU = primary healthcare unit.

* Information relating to the variables was not always available; the percentages were calculated by taking n as the number for which information was present.

## DISCUSSION

The employment/unemployment situation is decisive in determining family stability. Unemployment worsens families' socioeconomic conditions and increases their vulnerability. This is significantly reflected in the care given to children and the provisions made for their basic needs. Unemployment leads to increased risk of mortality, and particularly post-neonatal mortality, which is more strongly influenced by socioeconomic conditions. This vulnerability becomes more marked when unemployment affects the head of the household, who in the great majority of cases is a male figure (father/provider). This increases the frequency of marriage breakdowns: "in the light of so many unfulfilled expectations: for the man, who feels he has failed, with his authority weakened through not being able to ensure food and shelter for his family; and for the woman, who sees her chances of having something through the marriage dissipate".^11,12^

In the present study, some differences were found in relation to work and income that indicated that the families of the children who died were in a disadvantaged situation. In relation specifically to income, the disadvantage was more marked among the children whose deaths occurred during the post-neonatal period. This has been observed in other studies, and it signifies that this component of infant mortality is subject to greater influence from the socioeconomic conditions.^13^ The descent into informal employment relationships brings other consequences, particularly lower access to private health plans. Most of these plans are funded by the company that employs the workforce. Among the families of the children who died during the post-neonatal period it was seen that they had lower access to private health insurance, thus indicating that although the people in these families might be in employment, their jobs offer few benefits added to their salaries.

Structural unemployment and the descent into informal employment relationships are some of the factors that cause families to become destabilized, through the increased stress caused by difficulties in individuals' and families' survival.^14,15^ This may be reflected in the behavior of the infant mortality coefficient in certain segments of the working class, particularly with regard to the post-neonatal component.

## ACCESS TO HEALTH SERVICES

Access to health services has a fundamental role in infant mortality investigations, since it constitutes an important element in the different social classes' broadly based consumption (education and the consumption of health and leisure services). The children of parents who have a low socioeconomic situation have less access to such services, even if they are located close to their home. The reasons for this are of a structural nature connected with the social class to which they belong: their parents' need to undertake long working days and to reduce the time they dedicate to broadly based consumption, because of their small share in the distribution of the nation's wealth.^16^

The influence of access to health services on the profile of infant mortality can be analyzed from the starting point of characterizing the prenatal care that was provided for the mothers whose children died before completing one year of age. The Brazilian Ministry of Health recommends that there should be six or more prenatal consultations, starting in the first trimester of pregnancy, with laboratory tests performed, and that these should be the indicators of quality in prenatal care.^17-20^ In our study, in both subgroups of infant deaths, the prenatal coverage was around 90%. Looking at this the other way round, we found that in the cases of 10% of the infant deaths that occurred in the municipality of Embu in 1996-97, the mothers had not had any prenatal consultations. The prenatal coverage rate was similar to what was found for the whole country (90.15%) and in the city of São Paulo (92.9%), in the same year.^21,22^

We defined adequate prenatal care as attendance at six or more consultations that started in the first trimester of pregnancy, with all the standardized examinations for the prenatal routine. It should be noted that the attendance and its starting date are indicators that, separately, evaluate the adequacy of the access to prenatal care. Thus, adequate prenatal care was obtained only in the cases of 35.5% (95% confidence interval, CI: 28.8-42.1) of the pregnancies in the population survey, with a similar pattern among the pregnant women with neonatal deaths and a higher proportion (44.8%) in the other subgroup of pregnant women. With regard to the access indicators, there were low percentages of pregnant women in the two mortality groups who had had six or more prenatal consultations: 46.5% in the subgroup of neonatal deaths and 51.8% in the subgroup of post-neonatal deaths, versus 66.7% in the population survey, which was a statistically significant difference. The proportion of the pregnant women in the neonatal and post-neonatal mortality subgroups who started their prenatal care within the first trimester was also statistically significantly lower than the proportion of the population survey group: 41.7% for those whose children died in the neonatal period and 40.0% for those whose children died in the post-neonatal period, versus 65% for the population survey (95% CI: 55.8-77.5). Differences in access that were associated with socioeconomic conditions have been described in other studies.^23^ Good-quality prenatal care reduces the complications during pregnancy and consequently the incidence of both neonatal deaths and the sequelae that would increase the infant's vulnerability and chances of dying before completing one year of life, even after surviving the neonatal period. Moreover, the conditions that favor low birth weight (access and socioeconomic conditions) persist within these families. After completing the period of hospitalization in a neonatal unit, these conditions will favor the onset of illnesses and even death during the first year of life. A connection between the prenatal care and the place where delivery took place, which is an important quality criterion,^24^ was not possible at that time since there was no maternity ward in the municipality.

Birth weight has been considered to be an important indicator of the population's health. Children with low birth weight (< 2500 g) present a greater risk of becoming ill and dying, and for this reason this factor is considered to be the most important single factor for infant survival.^25^ Among the children who died during the neonatal period in Embu in 1996-97, the frequency of low birth weight was found to be 63.9% and, among those who died during the post-neonatal period, the frequency was 36.9%. On the other hand, in the population survey, the frequency was 8.4%. It is emphasized that, over recent decades, the frequency of low birth weight has presented an apparently paradoxical pattern. There has been a reduction in the differences between the frequencies of low weight, with stagnation or even increases in this indicator in more developed regions.^26,27^ Some hypotheses have been put forward for explaining this, and these have been greatly studied. Better control during prenatal care, with greater possibilities for early diagnosis of high-risk situations, may have given rise to premature births that previously might have been stillbirths. Moreover, mothers in older age groups, especially in the population segments with greater income and schooling levels, have presented greater frequencies of arterial hypertension or other complications, with greater risk of fetuses suffering from intrauterine growth restriction.

This situation of low birth weight may also be attributed to early delivery without indication for this, through elective cesareans. In developed countries, the advance in technology aimed at reducing the incidence of stillbirth has also been accompanied by increased frequency of low birth weight and prematurity. In Brazil, the use of this technology may partially explain such increases. Analysis of the Pelotas cohorts^28^ has demonstrated that the increased incidence of low birth weight occurred in both cesarean and vaginal deliveries, and therefore there may be different causes for these two situations. The authors of the Pelotas study observed other changes over that period that might also be responsible for increases in low birth weight and prematurity, such as greater numbers of mothers without partners and greater numbers of teenage pregnancies.

At the primary healthcare units in Embu, the enrollment rate among the children who died after the neonatal period was 60%. This profile was very different to what was found for the population survey group, in which around 80% were enrolled in primary healthcare units, of whom 87.6% were enrolled in primary healthcare units in Embu.^29^ In addition to the difficulties these families had in accessing the health services, another possible explanation is that these children had chronic diseases and for this reason were using services outside of the municipality, which consequently would mean that they did not form any linkage with the primary healthcare units that would ensure more integrated follow-up for them.

From the post-neonatal deaths, for which the main causes related to avoidable diseases such as pneumonia and diarrhea, it could be seen that there was a high percentage of deaths at home, this is an expression of the difficulty in accessing the health services among the families with worst socioeconomic conditions.^17,30^

The health services, which should have a positive impact on individuals' health and perform a role of diminishing suffering, often have a negative impact because of the low quality of the care provided. This was observed in the cases the children who died at home during the post-neonatal period and the children who died after the neonatal period, in hospitals or emergency services. These situations show how the local healthcare system and the more complex levels of care were disjointed at the time of this study. They denote a lack of full provision of care for children, since hospital actions were disconnected from the outpatient actions within the municipal healthcare units. It also shows the low quality of medical care, which is an important risk factor for infant mortality.^31,32^

Precarious employment situations and low income levels translate into serious deficiencies in basic living conditions: food, housing, basic sanitation, education and health. The response to this picture has been to prioritize policies that focus on the problems, within the context of social reforms in Latin America.^33^ This has meant the adoption of compensatory measures for the effects of structural adjustments on populations that were already structurally vulnerable, to the detriment of the universalization of social rights. Measures aimed at reducing the mortality coefficients have intrinsic value since they free thousands of children from death; however, they do not change the essence of the social inequality and do not promote positive discrimination.^34^

The Infant Mortality Committees that are organized within municipalities and health districts in accordance with technical regulations from the Ministry of Health work using the concept that infant death is a sentinel event and therefore that all deaths that are considered avoidable must be investigated and analyzed. This model is structured according to the principles of "health surveillance". This is taken to consist of practices within the logic of the health model that are aimed towards facing problems that are selected because of their high impact on the living conditions of population groups.^35^ For these committees to expand the possibilities for their actions, it is fundamental for them to adopt interdisciplinary and intersectoral practices. Their frame of reference should not be restricted to the field of healthcare but, rather, it should include the need to engage in dialog with other fields of knowledge such as human and social sciences.^36^ It is therefore not enough to improve the access to health services and put forward programs that may change the coefficient of infant mortality: it is also necessary for other sectors of the municipal or state administration to present proposals that aim towards including these families in the world of work, school and leisure, as an alternative to the policies currently implemented, which are solely compensatory.

## CONCLUSIONS

The employment/unemployment situation was decisive in determining the degree of family stability and vulnerability to the occurrence of infant deaths, in addition to the access to and quality of healthcare services.
